# Regulation of ER-Golgi Transport Dynamics by GTPases in Budding Yeast

**DOI:** 10.3389/fcell.2017.00122

**Published:** 2018-01-24

**Authors:** Yasuyuki Suda, Kazuo Kurokawa, Akihiko Nakano

**Affiliations:** ^1^Live Cell Super-Resolution Imaging Research Team, RIKEN Center for Advanced Photonics, Saitama, Japan; ^2^Laboratory of Molecular Cell Biology, Faculty of Medicine, University of Tsukuba, Tsukuba, Japan; ^3^Department of Biological Sciences, Graduate School of Science, The University of Tokyo, Tokyo, Japan

**Keywords:** ER, Golgi, yeast, Sar1, Rab, Arf, COPII, COPI

## Abstract

A large number of proteins are synthesized *de novo* in the endoplasmic reticulum (ER). They are transported through the Golgi apparatus and then delivered to their proper destinations. The ER and the Golgi play a central role in protein processing and sorting and show dynamic features in their forms. Ras super family small GTPases mediate the protein transport through and between these organelles. The ER-localized GTPase, Sar1, facilitates the formation of COPII transport carriers at the ER exit sites (ERES) on the ER for the transport of cargo proteins from the ER to the Golgi. The Golgi-localized GTPase, Arf1, controls intra-Golgi, and Golgi-to-ER transport of cargo proteins by the formation of COPI carriers. Rab GTPases localized at the Golgi, which are responsible for fusion of membranes, are thought to establish the identities of compartments. Recent evidence suggests that these small GTPases regulate not only discrete sites for generation/fusion of transport carriers, but also membrane dynamics of the organelles where they locate to ensure the integrity of transport. Here we summarize the current understandings about the membrane traffic between these organelles and highlight the cutting-edge advances from super-resolution live imaging of budding yeast, *Saccharomyces cerevisiae*.

## Introduction

The earliest stages of intracellular membrane trafficking are comprised of interactions between the endoplasmic reticulum (ER) and the Golgi apparatus (Golgi). These organelles are not stable but rather exist as transient compartments in which size is maintained by the concomitant influx and efflux of membrane material that is regulated by the secretory pathway. Small GTPases are the key regulators of such membrane trafficking in most eukaryotic cells and, as they function as molecular switches to regulate both generation and fusion of transport carriers, they are also able to perform homeostatic compensation for their host organelles.

## Sar1 assembly around ERES

The ER is the first organelle where secretory cargo proteins are incorporated into its lumen to await the synthesis, modification and folding of nascent polypeptides for delivery to the Golgi apparatus. Delivery of these cargo proteins from ER to the Golgi is facilitated by COPII vesicles consisting of the inner coat Sec23-Sec24 and outer coat Sec13-Sec31 complexes. Recruitment of these coat proteins is triggered by the activation of Sar1 GTPase by its guanine nucleotide exchange factor (GEF) Sec12, an ER-resident membrane protein (Barlowe and Schekman, 1993). The amphipathic helix of active Sar1 is exposed and associates with the ER membrane to drive recruitment of the inner coat complex by direct binding with Sec23 together with the enrichment of cargo protein by the formation of the pre-budding complex. Subsequent recruitment of the outer coat complex through the interaction of Sec31 with Sar1-Sec23 leads to the caged structure of the COPII lattice which forms the vesicles (Sato and Nakano, 2007).

**Figure 1:**
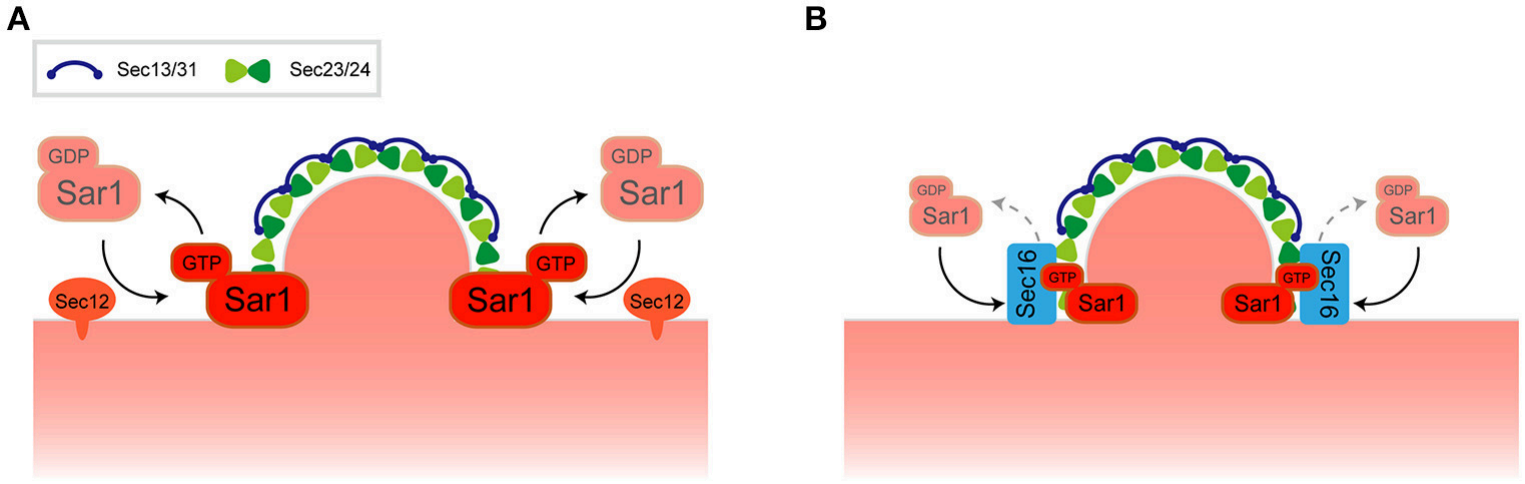
Regulation of COPII transport carrier at ERES. **(A)** A model for COPII transport carrier formation. **(B)** Recruitment of Sec16 to ERES regulates Sar1 localization at the rim of COPII-coated membrane.

ER-exit sites (ERES), or transitional ER sites, are the sites for COPII carrier accumulation on the surface of the ER (Palade, 1975; Orci et al., 1991). In budding yeast, the network-like structure of the ER is connected to the nuclear membrane and expands underneath the plasma membrane. This unique structure is facilitated by the reticulons in concert with Yop1/DP1-family curvature-stabilizing proteins and also by the action of an atlastin family protein-driven membrane fusion (Voeltz et al., 2006; Hu et al., 2009). Detailed ER structural composition is due to the connection of a branching tubule network with flattened, fenestrated sheets (West et al., 2011). Understanding of the structural organization of ERES has been recently assisted by the help of live-cell observations (Okamoto et al., 2012; Yorimitsu and Sato, 2012) and findings indicate that it is not uniformly observed in the surface of ER but has a relatively restricted formation. In the budding yeast *S. cerevisiae*, ERES marked by Sec13 showed numerous punctate structures on the network-like ER structure (Shindiapina and Barlowe, 2010; Okamoto et al., 2012). Moreover, the restrictive distribution of ERES is predominantly observed at the high-curvature domain of the ER membrane (Okamoto et al., 2012). Observations of the reticulon Rtn1 showed a non-uniform, restricted distribution along the ER and significant accumulation at both the tubules and sheet rims (De Craene et al., 2006; Voeltz et al., 2006; Okamoto et al., 2012). Localization for Rtn1 and ERES marked with Sec13 were significantly overlapped, suggesting ERES preferentially organized at the ER high-curvature domain (Okamoto et al., 2012). This domain might have both positive and negative curvatures that consist of various, differently shaped lipids that form the saddle-like domains of ER membranes. What is the underlying molecular mechanism of this non-uniform distribution of ERES within the ER high-curvature domain? The generally accepted idea is that construction of the COPII transport carrier is initiated by the activation of Sar1 by the ER membrane-localized GEF (Sec12) thereby causing Sar1-GTP recruitment of Sec23/24 to form the pre-budding complex (Figure 1A). One possible reason for the preference of ERES to reside in the ER high-curvature domain could be based on the structural preference of the Sar1-Sec23/24-cargo protein pre-budding complex that might recognize a specific domain of the ER (Bi et al., 2002). Another possibility includes the regulation of Sar1 GTPase activity. Upon the activation of Sar1 by Sec12, the Sar1 amphipathic helix inserts into the ER membrane and this helix of Sar1-GTP may be favored by high-curvature, tubule-enriched domains within the ER. Indeed, reticulon and Yop1/DP1 mutations that result in a compromised ER tubular network cause accumulation of ERES at the residual high-curvature domain of the edge of unfenestrated peripheral ER sheets (Okamoto et al., 2012). Insertion of the Sar1 amphipathic helix generates deformation of ER membrane that is proposed to mediate the formation and scission of COPII transport carriers (Lee et al., 2005). It is also argued that a positive feedback loop exists between the curvature, Sar1 GTPase, and membrane deformation, as Sar1 GTPase is stimulated by the local curvature of the ER membrane (Hanna et al., 2016; Jarsch et al., 2016). Contrary to the preceding case, COPII carrier formation based on this Sar1 positive feedback loop may contribute to the generation of high-curvature domains within the ER membrane.

Live cell imaging suggests that the localization of Sar1 is not strictly confined to ERES but is rather distributed throughout the ER membrane together with some punctate accumulations near ERES. Detailed inspection of the relationship between Sar1 and Sec13 revealed that Sar1 accumulates at the rims of COPII-coated membranes and is also excluded from the rest of Sec13-labeled COPII-coated membranes (Kurokawa et al., 2016). Sec16, a component of ERES, could be the key player for the restrictive accumulation of Sar1 at the rim. It has been proposed that *Pichia postoris*, a different budding yeast, Sec16 functions to maintain a ring of Sar1 at the edge of the COPII-coated membrane (Bharucha et al., 2013). Recruitment of Sec16 to ERES is dependent on the Sar1 GTPase cycle, thereby inhibiting Sec23-mediated GTP hydrolysis by Sar1 (Kung et al., 2012; Yorimitsu and Sato, 2012; Iwasaki et al., 2017; Figure 1B). These observations have posited Sec16 as the regulator of COPII turnover. Both enhancement and inhibition of Sar1 GTPase activity is important for Sar1 at the rims of COPII-coated membranes as defects in either function compromises the specific localization of Sar1 (Kurokawa et al., 2016). It is noteworthy that in *P. pastoris*, ERES is also found at flat-surface of the nuclear membrane (Mogelsvang et al., 2003). *P. postoris* Sec16 has been shown to recruit Sec12 at ERES, which counts as an additional layer of Sar1 regulation at ERES (Montegna et al., 2012). Thus, not only Sar1 activation, but also Sar1 GTPase cycling, facilitates COPII carrier formation within the ERES, thereby limiting the specific localization of Sar1. In any case, further investigation may reveal how the roles of the high-curvature domain, its lipid composition, and the regulation of Sar1 GTPase activity are interconnected with each other and how this determines the significant preference for ERES to reside within the ER.

## Association of ERES and the Golgi

The greater part of eukaryotes exhibit a tight *cis* to *trans* association between each compartment of the Golgi cisternae to form the stacks. Golgi stacks are functionally interconnected with ERES by the formation of the ER-Golgi intermediate compartment (ERGIC) or vesicular tubular clusters (VTCs) (Budnik and Stephens, 2009; Johnson et al., 2015). These organelles are structurally distinct from the ER and the Golgi and are generated by the homotypic fusion of COPII vesicles (Hobman et al., 1998; Xu and Hay, 2004) whereas in budding yeast, each *cis* to *trans* cisterna of the Golgi is dispersed throughout the cytoplasm (Losev et al., 2006; Matsuura-Tokita et al., 2006). How, then, is the faithful delivery of the cargo proteins from the ER to the Golgi established? Admittedly, free COPII-vesicles are less visible in electron microscopic studies than COPI- and clathrin-coated vesicles within the cells. A reason for this observation could be that transport by COPII-vesicles is over a relatively short distance and is a short-lived event, making free COPII vesicles hard to observe in electron microscopic observations. In *S. cerevisiae*, COPII transport is immediately completed upon generation at ERES by reaching to the floating cisternae in the cytoplasm. Indeed, we have recently shown that the *cis*-Golgi, but not *trans*-Golgi, frequently approaches toward ERES in living cells (Kurokawa et al., 2014). It is considerably safer for the COPII vesicles to be captured by the Golgi rather being released into the cytoplasm. The dynamic approach motion of *cis*-Golgi, called “hug-and-kiss” action, was shown to be disturbed when efficient transport was compromised by glucose depletion or incubation at restrictive temperatures in *uso1-1* cells (Kurokawa et al., 2014). Faithful transportation from the ER to the Golgi in budding yeast is governed by dynamic and temporal formation of an ER-*cis-*Golgi unit, in which Sar1-driven, COPII carrier formation at ERES is captured by the cisternae of *cis-*Golgi. Thus, the ER-*cis-*Golgi unit in budding yeast is essentially equal to the ER-ERGIC found in mammals.

## The Golgi apparatus

As the Golgi is regarded as a transient organelle, its integrity is ensured by the turnover of membrane source within the intracellular trafficking pathway and secretory cargo proteins are modified, delivered and sorted at the level of the Golgi. Out of the numerous analyses that attempt to explain intra-Golgi transport, the widely accepted model is cisternal maturation (Nakano and Luini, 2010; Glick and Luini, 2011). Cisternal maturation was first observed directly in live cells of the budding yeast *S. cerevisiae* (Losev et al., 2006; Matsuura-Tokita et al., 2006). This process is accomplished by the COPI-dependent retrograde transport of Golgi-resident proteins, thus the GTPases that regulate COPI-carrier formation and fusion are important in this regard. Since GTPases act as the switch for membrane traffic within the cells, morphology and integrity of the Golgi are regulated by the activity of GTPases specifically localizing at the Golgi. Arf GTPases and some Rab GTPases are specifically targeted to the Golgi apparatus in budding yeast. Arf GTPases function mainly in the generation of transport carriers, such as COPI and clathrin, whereas Rab GTPases function in the fusion process of these transport carriers. Glick and colleagues have proposed that Golgi functions can be sorted into several functional categories as they transit from carbohydrate synthesis to carrier formation stages (Papanikou and Glick, 2014). Intra-Golgi transport mediated by COPI is thought to act in the carbohydrate and carrier formation stages while transport machinery such as clathrin directs the traffic toward post-Golgi organelles and the plasma membrane. Thus, the functional switch from carbohydrate synthesis to carrier formation within the Golgi might determine the identity of the cisternae. In fact, COPI co-localizes with *cis*-Golgi-resident protein and clathrin heavy chain (Chc1) co-localizes with a *trans*-Golgi/TGN marker, suggesting that the temporal distributions of COPI and clathrin within the Golgi segregate in the course of Golgi maturation (Daboussi et al., 2012; Papanikou et al., 2015). In line with this idea, upstream regulatory mechanisms to facilitate these distinct carriers' formation within the Golgi could determine the definitions of these stages and presumably mediate cisternal maturation of the Golgi.

## Transition of Rab GTPases in the Golgi

**Figure 2:**
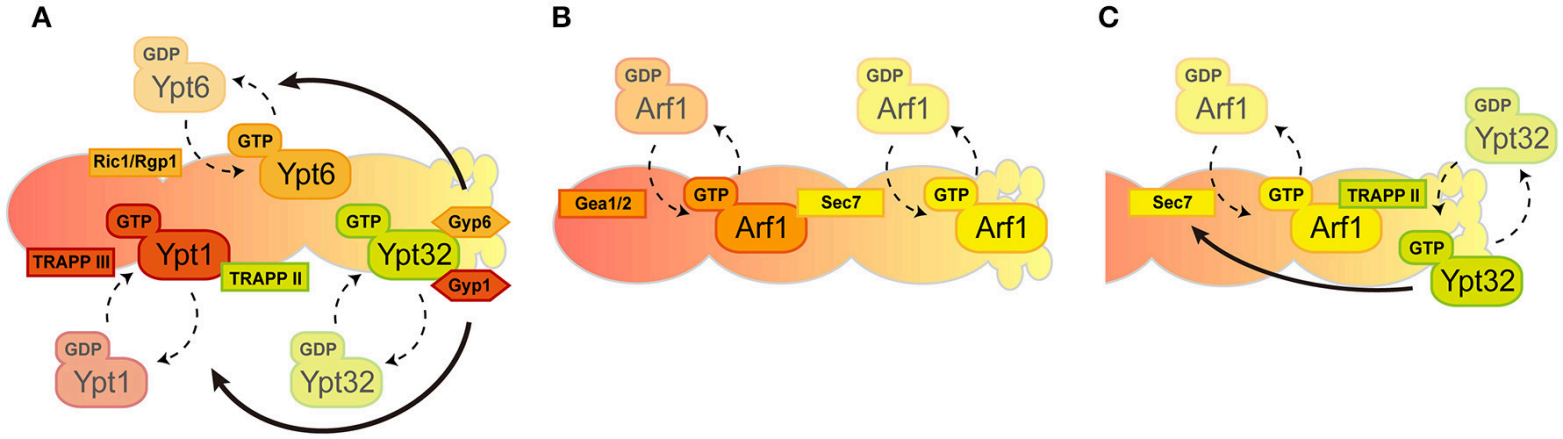
Spatio-temporal regulation of GTPases in the Golgi. The Golgi compartments are shown in a *cis-*to-*trans* orientation going from left to right. **(A)** Consecutive recruitment of Rab GTPases created by GEF and GAP cascade. **(B)** A series of Arf1 activation by local GEFs. **(C)** Positive feedback loop between Arf1 and Ypt32 through their GEFs in *trans-*Golgi/TGN.

Rab GTPases punctually and spatially regulate specific and distinct membrane traffic within the Golgi that is coordinated by GEF and GAP (Segev, 2011). These Rab GTPases appear and act consecutively during the maturation of the Golgi: Ypt1 appears firstly at the *cis*-Golgi, Ypt6 is then recruited and lastly Ypt31/32 accumulates at the *trans*-Golgi/TGN wherein Ypt1 and Ypt6 no longer exist. Mutually exclusive localization of these Rab proteins are facilitated by the GEF and GAP cascade in which early-acting Ypt1 recruits the GEF for Ypt32 as an effector then Ypt32 recruits the GAPs for Ypt1 and Ypt6 as effectors (Figure 2A; Rivera-Molina and Novick, 2009; Suda et al., 2013). Note that the favorable model that has been most recently mentioned includes only two TRAPP complexes act as GEFs for Golgi Rab GTPases in yeast; TRAPPIII for Ypt1 and TRAPPII for Ypt31/32 (Thomas et al., 2017). Rab GTPases recruit tethering proteins as effectors and mainly function in the fusion process of transport carriers as Ypt1 recruits the conserved oligomeric Golgi (COG) complex for the fusion of COPI vesicles and Ypt6 recruits the Golgi associated retrograde protein (GARP) complex for the endosome-derived vesicles (Siniossoglou and Pelham, 2001; Suvorova et al., 2002). We therefore reasoned that the transition of Rab GTPases could drive Golgi maturation but results showed that the speed of Golgi maturation was affected but not disturbed in Rab transition failure mutants (Suda et al., 2013). This suggests that Rab GTPase-mediated membrane trafficking is most likely a secondary regulatory mechanism for Golgi maturation. Another study of Rab GTPase function in Golgi morphology showed that the activation of Ypt1 or Ypt31 resulted in increased rates of cisternal progression on the compartments they reside, suggesting the importance of Rab GTPases for Golgi maturation (Kim et al., 2016). This all leads to the critical question: how does the maturation of the Golgi proceed? Recent evidence suggests that maturation could rely on the connection of Rab GTPases with the regulation of other GTPases (such as Arf1) through their GEFs in the Golgi (see below).

## Regulation of Arf1 GTPase activity in the Golgi

Golgi-associated Arf GTPase recruits effectors, including COPI coat proteins and clathrin adapters. The Glick group showed that the Golgi enlargement and defects in both the speed and efficiency of the *cis*-Golgi maturation in Arf1-depleted cells is presumably caused by Arf1-mediated recruitment of COPI-coat proteins (Bhave et al., 2014). Indeed, COPI loss-of-function status compromises the integrity of the Golgi maturation, corroborating the key role of Arf1 in the Golgi trafficking (Papanikou et al., 2015; Ishii et al., 2016).

In general, the Golgi functions are composed of two discrete stages: “carbohydrate synthesis” in which Golgi-resident glycosylation enzymes are recycled by COPI and “carrier formation” is mediated by clathrin-coated vesicles. COPI vesicles are generated mainly from *cis-*Golgi cisternae while clathrin vesicles are generated only from TGN and not earlier cisternae. Coat proteins for these transport carriers are recruited to the Golgi by a single Arf1 GTPase. This raises the question of how the singular activity of Arf1 mediates multiple effector recruitment within the Golgi. One possible underlying mechanism to answer this question is the existence of multiple GEFs for Arf1 in the Golgi. Three Arf-GEF proteins have been shown in yeast to catalyze the GTP binding of Arf1-containing GBF family proteins: Gea1/2, BIG family protein Sec7 (Wright et al., 2014). These Arf-GEFs could facilitate the coordination of specific Arf1 GTPase activity toward multiple downstream effectors. Gea1/2 and Sec7 are thought to have different preferences for their localization within the Golgi as Gea1/2 specifically resides at *cis*-cisternae and Sec7 localizes mainly at the *trans*-Golgi/TGN. Recruitment of Sec7 to the later-compartment of the Golgi is governed by autoinhibition, the positive feedback-loop between Arf1 and Sec7, and by one of the Rab GTPases Ypt1 and Arf-like GTPase (Arl1) (Richardson et al., 2012; McDonold and Fromme, 2014). Gea1/2 was shown to interact with several proteins such as Sec21, a COPI coat protein, and Drs2, a *trans*-Golgi/TGN-localized aminophospholipid flippase, thus their specific localization within the Golgi was enigmatic (Chantalat et al., 2004; Deng et al., 2009; Tsai et al., 2013). However, recent findings have shown a preference of Gea1/2 for the *cis*-Golgi (Figure 2B; Gustafson and Fromme, 2017). The preference of these Arf-GEFs to localize at distinct compartments within the Golgi mirrors the surface environment of the Golgi membrane. Anionic lipids such as phospatidylserine (PS) and phosphatidylinositol-4 phosphate (PI4P) are enriched from *cis*-to-*trans* on the Golgi surface and governed by the activities of Drs2 and Pik1, respectively (Walch-Solimena and Novick, 1999; Natarajan et al., 2004; Strahl et al., 2005). It should be noted that Arl1-mediated membrane remodeling through Drs2 is also important in this step (Yu and Lee, 2017). Sec7 prefers the anionic surface of the membranes to facilitate maximum catalytic activity toward Arf1, conversely, Gea1/2 prefers more a neutral environment of the membranes (Gustafson and Fromme, 2017). In addition, the interactive feedback loop between Arf1 and Rab GTPase has been also shown at the level of *trans*-Golgi/TGN. Recruitment of TRAPPII components that function as a GEF complex for Rab GTPases at the *trans*-Golgi/TGN, such as Ypt31/32, is facilitated by the anionic surface of *trans*-Golgi/TGN and Arf1-GTP (Thomas and Fromme, 2016). Initial recruitment of Sec7 is governed by Ypt1, Arf1, and Arl1, followed by Ypt31/32, stimulates the activity of Sec7 toward Arf1 (McDonold and Fromme, 2014). These studies therefore establish the model for the regulatory circuitry of Arf1 GTPase and Rab GTPases within the Golgi (Figure 2C). In this scenario, specific regulation of Arf1 (for the generation of COPI and clathrin vesicles) and Rab GTPase activity (for the fusion of these transport carriers), together with the lipid composition of surface membranes within the Golgi, may drive the functional shift from the COPI-generated, carbohydrate synthesis stage to the clathrin-mediated, carrier formation stage.

In summary, the GTPase cycling of Sar1, Arf1, and Rab GTPases are tightly regulated by their specific mediators to not only facilitate the formation/fusion of their own transport carriers but also to regulate the integrity of the specific compartment within their resident organelles, thereby facilitating fundamental membrane trafficking in the early secretory pathway.

## Author contributions

All authors listed have made a substantial, direct and intellectual contribution to the work, and approved it for publication.

### Conflict of interest statement

The authors declare that the research was conducted in the absence of any commercial or financial relationships that could be construed as a potential conflict of interest.
